# Measuring Motivation for Appetitive Behaviour: Food-Restricted Broiler Breeder Chickens Cross a Water Barrier to Forage in an Area of Wood Shavings without Food

**DOI:** 10.1371/journal.pone.0102322

**Published:** 2014-07-28

**Authors:** Laura M. Dixon, Sarah Brocklehurst, Vicky Sandilands, Melissa Bateson, Bert J. Tolkamp, Rick B. D'Eath

**Affiliations:** 1 Animal and Veterinary Science, SRUC, Edinburgh, United Kingdom; 2 Biomathematics and Statistics Scotland, Edinburgh, United Kingdom; 3 Center for Behaviour and Evolution, Institute of Neuroscience, Newcastle University, United Kingdom; Liverpool John Moores University, United Kingdom

## Abstract

Broiler breeders (parents of meat chickens) are selected for fast growth and become obese if fed ad libitum. To avoid this and maintain good health and reproductive ability, they are feed restricted to about 1/3 of what they would eat ad libitum. As a result, they experience chronic hunger and exhibit abnormal behaviour patterns that may indicate stress and frustration. One approach to measuring hunger is to observe how much birds will work, such as pecking a key, for access to more or different types of food. However, the sight, smell, and feedback from consumption of the feed reward changes the context and may artificially raise feeding motivation. To avoid this, we tested broiler breeders in an apparatus in which they could work for access to a wooden platform covered in wood shavings by crossing a water runway which increased in length and depth in 8 successive tests. In the wood shavings area, they could perform exploratory and foraging behaviour (the appetitive phase of feeding) but were never rewarded with feed. Sixty birds were divided into three feed quantity treatments: commercial restriction (R), and twice (2R) or three times (3R) this amount. Overall, birds fed R worked harder to reach the wood shavings area (reached it in a larger number of tests) than 2R and 3R birds (P<0.001). More restricted birds took less time to reach the area (P<0.001, R<2R<3R) and spent more time foraging while there (P<0.001, R>2R>3R). This indicates that restricted-fed birds were hungry and willing to work for the opportunity to forage even though food was never provided, suggesting that their motivation to perform the appetitive component of feeding behaviour (foraging/food searching) was sufficient to sustain their response. Thus food restriction in broiler breeders is a welfare concern. However these methods could be used to test alternative feeding regimes to attempt to find ways of alleviating hunger while still maintaining healthy growth and reproduction in these birds.

## Introduction

Ethical concerns over animal welfare arise because of the belief that animals can experience and suffer from negative mental states [1], [2]. Although we cannot be sure whether animals consciously experience emotions, we can nonetheless investigate the physical and behavioural outward signs of emotion in animals [1], [3]–[6]. Emotional states accompany situations that are rewarding (positive emotions) or punishing (negative emotions), so investigation of animal motivation is an important behavioural approach in animal welfare science [1], [7]. Typical methods to measure motivation require animals to overcome a cost to gain access to a resource: for example, the animal is asked to work by e.g. repeatedly pressing a lever or pushing through a weighted door to obtain an immediate reward (operant or consumer demand tests, e.g. [1], [8]–[12]). Animals that are willing to work hard to obtain a resource are said to be motivated to access that resource. Therefore, they would have improved welfare if provided with, and decreased welfare if denied, that resource.

There are some difficulties involved in measuring motivation for a resource (e.g. [13], [14]). As most motivation tests provide the resource either as part of the test or as the reward for completing the task (e.g. [8], [15], [16]), sights and smells and feedback from the resource may initially be artificially raising the animal's motivation for that resource while without these cues motivation may remain low (e.g., out of sight is out of mind, [17]).

The present study focuses on motivation for food in broiler breeders. Broiler breeders (the parents of broiler chickens) grow rapidly, become overweight, and suffer from a number of health problems associated with obesity resulting in high mortality if they are fed to appetite [18]. To prevent this, they are ration-fed, resulting in food restriction which can be as severe as 33% of what they would eat *ad libitum* [19], [20]. This gives rise to the welfare concern that they may be suffering from chronic hunger (reviewed by [21], [22]). The ‘broiler breeder paradox’ [23] is that it is difficult to feed them so that they are both ‘healthy’ and ‘have what they want’, which Dawkins [24] has proposed as the key determinants of good animal welfare.

Food restricted broiler breeders show increased general activity and in particular foraging activity such as scratching and pecking. This increased foraging activity can also be expressed in abnormal ways, such as through spot pecking and polydipsia or water spillage [25]–[29].

In addition to these observations, feeding motivation in broiler breeders has also been tested by presenting extra food, for example in a preference or operant conditioning task [30], [31] or a relatively short rate of eating task using familiar or novel food [29], [32], or in a longer-term compensatory feeding task which assesses the extent of previous food restriction by measuring *ad libitum* food intake over 22 days [33], [34]. Taken together, this evidence indicates that food restricted birds will be chronically hungry and experiencing stress, which has a negative impact on bird welfare [19], [21], [25], [27], [28]. The use of food in these feeding motivation tests raises a number of difficulties (see [35] for a detailed critique). The presentation of additional food in these tasks changes the context for the animal from having to cope because it has no access to food to being aware that additional food is available (even if they must work to get it). Thus, (i) the potential to obtain the resource is in itself likely to increase motivation and (ii) once the resource has been used motivation may be increased as a result of positive feedback [36], until at least a state of satiety is reached. When birds on different feed treatments are compared, there are additional difficulties of interpretation, and this raises the question of whether the usual treatment food, or a single food type common to all treatments should be used in the test situation.

In the present study, we propose to avoid the difficulties with food-based tests by measuring motivation to access a location where exploratory and food-searching (foraging) behaviour is possible but in the absence of a food reward. Many behaviours, including feeding, contain an appetitive phase which involves the searching phase of a behaviour sequence (exploration, and searching for food; foraging) and indicates the need or motivation to achieve a certain goal. Consummatory behaviour (e.g. eating) follows appetitive behaviour and is the achievement (‘consummation’) of the goal or behaviour needed to help satisfy the motivation [37], [38]. The appetitive phase of behaviour continues until the goal is reached but unless the motivation is fulfilled, performance of the appetitive behaviour should persist (unless it continues to a point where other motivations become more important) [39], [40]. Thus feeding motivation could be estimated by measuring motivation to perform the appetitive phase of feeding behaviour, i.e. foraging.

Another difficulty with tasks to measure feeding motivation concerns the nature of the cost imposed. Animals may find it easier to associate a task involving pecking with access to food, rather than with another non-food resource [1], [11]. Key pecking at increasing schedules (i.e. more pecks per reward) were used by Savory et al [41] and Savory & Lariviere [42] to show that food-restricted broiler breeders were 3.6 times more motivated for food than *ad libitum* fed birds which had been food deprived for 72 hrs. This striking demonstration of the high feeding motivation resulting from chronic food restriction is nonetheless open to the criticism that hungry birds increase their pecking anyway, so is increased pecking really a cost, or just an expression of this? More ‘natural’ costs such as squeezing through a narrow gap [43], or pushing through a weighted door [9], [10] or walking a long way [44] overcome this difficulty and also have the advantage that they require little or no learning [43]. In the present experiment we chose to use the natural cost of walking through water, which hens find aversive [45]–[47]. Water also has the advantage that the cost can be varied by increasing the length and depth of the water.

In earlier experiments we had found that broiler breeders were motivated to forage in areas to which they had intermittent access [14]. In the present study we designed a task in which broiler breeder female chickens were tested for their motivation to access a platform covered in wood shavings which was only accessible during the tests and never (at any stage of training or testing) contained a food reward. We imposed a ‘natural’ cost on access to the wood shavings area using a water runway which increased in length and water depth with each test. Birds were allowed 10 minutes from the start of the test to reach the wood shavings area and given a further 5 minutes in the apparatus if they did reach it. Birds (n = 20 per treatment) were given one of three feed treatments that differed in food quantity: the industry recommended restriction level (R), or two (2R) or three (3R) times this amount. It was predicted that birds fed smaller food portions would be more motivated to reach the wood shavings area, as indicated by i) a higher proportion of birds crossing the water at each cost. In the language of ‘animal economics’ which can be applied to these types of experiment, this would result in a greater ‘maximum price paid’ (reservation price [9], [10]) to reach the wood shavings area. We also predicted that more food restricted birds would show ii) a less rapid decline in birds crossing the water as it gets longer and deeper; iii) a shorter average time taken to cross the water and iv) a longer average time spent foraging once they reach the wood shavings area.

## Materials and Methods

### Ethical Considerations

Birds never had their water intake restricted and were housed on a bedding of wood shavings to provide comfort, insulation and allow for dustbathing behaviour. Food restriction is likely to result in hunger, but research into animal welfare problems often faces the difficulty that the problem must be recreated in the laboratory in order to study it. At any one time there are an estimated 7.5 million broiler breeder chickens in the UK alone [32], [49] all of which are restricted-fed (R) during rearing. The levels of food restriction we imposed were similar to or less severe than that used routinely in the poultry industry, with birds receiving at least the industry recommended level (R) of food or some multiple of this (2R or 3R). *Ad libitum* fed broiler breeders can suffer from health problems and mortality [18], and the ration of 3R birds in our study was close to *ad libitum* [50]. However, the experiment was ended when birds were 11 weeks old, at which age they were still active and healthy. All procedures in this experiment were carried out under Home Office Licence and with the SRUC Animal Experiment Committee's approval; birds were checked on a minimum of three times per day.

### Animals and Housing

Sixty non-beak trimmed Ross308 broiler breeder female chickens were received from Aviagen (Stratford, UK) as day old chicks, and were reared according to the commercial recommendations for housing, lighting, temperature and nutrition for this genotype: the Ross308 parent stock guidelines [48]. They were housed in floor pens (1.0×2.0 m) covered in wood shavings until 4 weeks of age. The lighting schedule for the first day was 23.5L:0.5D hours light:dark, which was then gradually reduced to 8L:16D over 10 days. Temperature decreased from around 30°C at bird level at one day old to around 20°C by four weeks of age. Chicks were given *ad libitum* water from bell drinkers and were fed chick starter crumbs for the first three weeks, chick starter pellets for the following three weeks and then grower pellets (all ABN, Cupar Mills, Fife) from the beginning of six weeks of age to the end of the trial. The feed was formulated in line with commercial broiler breeder standards. Food was provided *ad libitum* for the first 7 days and then in restricted amounts given at 9:00 am each day that were gradually increased from 26 to 44 g per bird per day by the beginning of the 5th week, as per the Ross308 parent stock guidelines [48].

**Figure 1:**
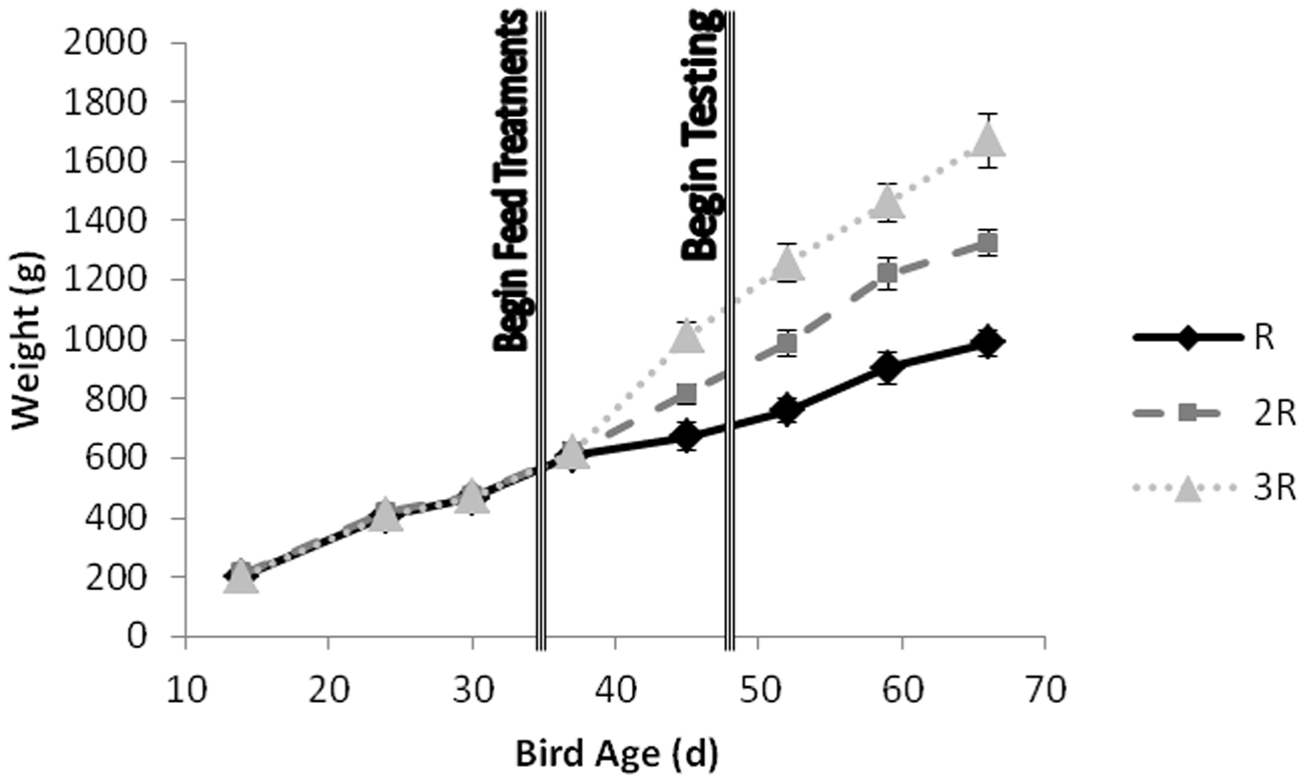
Bird weights for the 3 feed treatments (mean ± SD) from 14 days of age to the end of the trial. Birds began different feed treatments at 35 days of age and testing began at 50 days of age.

At 4 weeks of age, all birds were weighed, wing tagged (10 mm×10 mm padlock-style tags, Roxan Developments Ltd., UK) and housed in groups of three in twenty-one floor pens (1.5×2.0 m) according to matched body weight. However for three of the pens only two birds were used for the trial, giving a total of twenty birds per feed treatment. Two birds of each group were randomly selected to be the ‘marked’ birds (marked with either blue or purple livestock spray before testing), while the other was unmarked during testing. Each pen was bedded with wood shavings and provided with water through cup drinkers. To minimise food competition, food was presented in four small (90 mm width ×75 mm height ×55 mm depth) semi-circular food cups, two on the left side of the pen and two on the right. Starting in the 5th week of age, birds began to receive the commercially recommended food quantity (R), or two (2R) or three (3R) times this amount, depending on their feed treatment (see below). All birds were weighed approximately weekly from age 2 weeks to the end of the trial (age 11 weeks) (Fig 1). Although it is difficult to make an exact comparison because of differences in feed, genotype and rearing method, the 3R treatment achieved food intake and growth rate close to that of ad libitum fed birds [41]. However, where an *ad libitum* fed bird would have constant access to feed, the 3R birds generally finished their meals within the light periods (8 hours). The R birds finished their rations in 15 minutes or less and the 2R birds finished in 40 minutes or less.

### Apparatus

**Figure 2:**
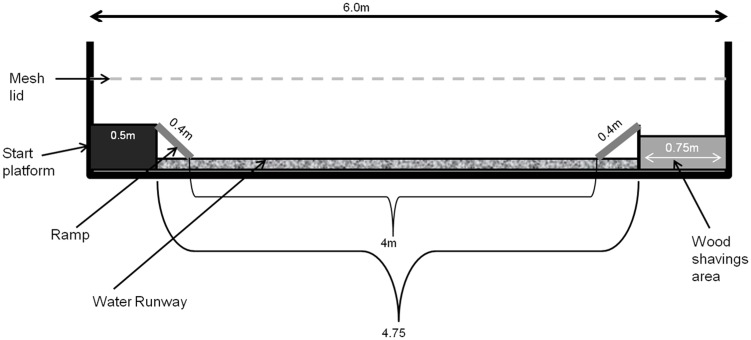
Diagram of the water runway apparatus. Birds were placed on the start platform and could chose to walk down the ramp into the water runway, up the second ramp and go onto the wood shavings area. The wood shavings area could be moved along the runway so that the distance travelled through the water could be increased up to a maximum of 4

Two identical sets of apparatus were used. Each apparatus was 6.0 m in length and was constructed with a wooden frame and wire mesh walls (Fig 2). Within this frame, the floor and sides to a depth of 130 mm of the apparatus were covered with a water proof tarp to allow for water to be added to the apparatus. At each test, birds began on a wooden start platform (0.5×0.5 m, height 0.14 m) at one end of the apparatus and they could progress to another wooden platform at the other end which constituted the wood shavings area (0.75×0.5 m, height 0.14 m). A 2 cm layer of wood shavings covered the wooden platform (retained at the front edge by a 10 mm high wooden batten) during training and testing but not habituation. The wood shavings area could be moved along the apparatus, allowing the runway length to vary. Ramps led from the start platform to the runway and from the runway to the wood shavings area (0.4 m each). This prevented the birds from having to jump into and out of the runway, in case they slipped in the water. Ramps were fitted with three lateral wooden battens 20 mmx20 mm in cross section and 0.15 m long, and placed at 0.08 m intervals to improve grip for the birds' feet. The ramps were always placed with a 25 mm overhang on the platforms to ensure ramp length stayed consistent throughout tests. Taking the ramps into account, the length of the runway between the two ramps that the birds needed to cross to reach the wood shavings area could range from 0 to 4 m.

A removable mesh lid that lowered the ceiling for the birds to just above head height was attached to the apparatus at the start platform and extended over the runway area to prevent birds from attempting to fly from the start platform to the wood shavings area. (Birds need extra height to lift off for flight and the lid prevented this). The lid was raised gradually from 0.15 to 0.25 m above the height of the start platform and wood shavings area as the birds grew so that they were always able to stand upright in the apparatus.

### Habituation

The chickens were housed in groups of three but were tested in the apparatus singly. Therefore the birds had to be comfortable enough on their own in the apparatus to perform the task. To help to achieve this, at 5 weeks of age, groups of three birds were placed in the apparatus (no water and no wood shavings) for 15 minutes to explore. This was repeated two more times for a total of three group habituation sessions to the apparatus. Next, each bird was placed individually in the apparatus to explore for 15 minutes and this training continued until the individual bird was walking around the apparatus and not making alarm calls: this took about three individual sessions.

### Training

Training began at 6 weeks of age. To begin, the apparatus did not have any water and wood shavings were spread on the wooden platform in the wood shavings area. There was *no* gap between the start platform and the wood shavings area (ramps were removed). Birds were left to explore the apparatus and to find the wood shavings. Once the birds reached the wood shavings area, they were allowed a further 5 minutes in the apparatus before the training session ended. The birds were given 10 minutes to find the wood shavings area. If they did not reach it in the 10 minutes given, they were placed in the wood shavings area for 5 minutes. Birds would not have progressed from this step until they reached the wood shavings area without assistance. However, in practice all birds were successful in their first training session with this apparatus set up.

Next the wood shavings area was moved 0.95 m from the start platform and the ramps were added, with a 20 cm gap between the bottom of the ramps. No water was in the runway and again birds were given 10 minutes to reach the wood shavings area and were left for a further 5 minutes after they did. Birds all reached the wood shavings on the first attempt, otherwise this training step would have been repeated until they did. Finally, this step was repeated but with enough water in the runway to just cover the birds feet (about 20 mm), as pilot studies suggested that birds found the initial appearance of the water aversive and began to alarm call when first exposed to the water. After all birds were successful in reaching the wood shavings area with 20 mm water in the runway, testing began. For this apparatus set up, all but 3 birds were successful in reaching the wood shavings area and the remaining 3 were given another training session the following day in which they all succeeded in reaching the wood shavings area.

### Testing

Testing began when the birds were 7 weeks of age and lasted 24 days. Each bird was tested on eight occasions in total, 3 days apart. For the first test, the wood shavings area was moved 1.25 m from the start platform, with 0.5 m between the bottom of the ramps and water was added to the runway. The room was heated to a constant 20–20°C, and the water was at ‘ambient temperature’ i.e. it was not heated separately. The same source of mains water was used to fill and refill the apparatuses. Because birds on the different feed treatments grew at different rates over the test, the water depth was proportional to mean leg length. To do this, the length of the birds' legs was measured from the ground to the top of the hock before each test for R, 2R and 3R fed birds. Over subsequent tests, the ‘cost’ of accessing the wood shavings area, in terms of water depth and length was increased in a stepwise manner: water was increased in increments relative to the average length of the birds' legs (Table 1). This resulted in water depth levels that ranged from 8 mm to 110–112 mm by the end of the eight tests. As the water depth increased with each test, the length of the runway was also increased by 0.5 m each time up to a maximum length of 4 m (measured from ramp end to ramp end; Table 1). The environment for the apparatuses was the same for all tests (between 20–22°C) and the water used to increase water depth came from the same source throughout the trial.

**Table 1:** Cost increases (runway length between the two ramps and water depth) with increasing test number for the 3 feed treatments.

	Test Number							
	1	2	3	4	5	6	7	8
**Runway Length (m)**	0.5	1.0	1.5	2.0	2.5	3.0	3.5	4.0
**Water Depth (percentage of leg length)**	17%	33%	50%	67%	83%	100%	117%	133%

Water depth is based on percentage of the mean length of the birds' legs, from the ground to top of the hock, for each food treatment. The initial water depth was 1/6^th^ of the average leg length and increments of 1/6^th^ were then made at successive tests so that in the last two tests the water was deeper than the birds' legs were long, meaning that her body was getting wet.

Each test lasted up to 15 minutes. A bird was placed on the start platform and given 10 minutes to get to the wood shavings area. Once a bird had reached the wood shavings area, she was allowed 5 minutes before the test was ended. If she did not reach it in the first 10 minutes, she was removed from the apparatus. These time limits were set for practical reasons (to reduce the time taken to test multiple birds) and to reduce the chances of extinction occurring.

### Experimental Design

The three different feed treatments (R, 2R and 3R) were allocated at the pen level in a randomized block design. The 21 home pens were divided into sets of three adjacent pens (7 spatial blocks), to which the three treatments were randomly allocated. Six of the blocks contained pens with 3 birds and the remaining block contained pens with 2 experimental birds resulting in 7 pens and 20 birds in each feed treatment. Three of the blocks of pens with 3 birds (selected at random) were tested on one set of apparatus, while the other three blocks were tested with the other set of apparatus. For the three pens with only two experimental birds one bird in each pen was tested on each apparatus. Birds within pens were allocated to one of two scheduling groups for which tests were staggered by one day in such a way that each scheduling group contained half of the birds. These two scheduling groups and the use of the two sets of apparatus were balanced with each other and as much as possible with spatial blocks and feed treatments.

### Measurements

For all tests, we recorded whether the bird reached the wood shavings area (defined by the bird having both feet on the platform) and the latency to reach the wood shavings area in cases where the bird was successful. For birds that reached the wood shavings area, their behaviour in the wood shavings area was also recorded to allow determination of whether the birds were using the wood shavings area for foraging. In detail, total durations that the birds spent in the wood shavings area standing and foraging (scratching and/or pecking), sitting and foraging (scratching and/or pecking), standing, sitting, walking or preening were measured over the 5 minutes from when they first reached the wood shavings area. Sitting was rare and so this was combined with standing for analyses and the behaviours preening and walking occurred at very low levels throughout all treatments so were not analysed further. Additionally, in the 5 minutes from when birds first reached the wood shavings area, birds were also able to leave the area and their movement was recorded so that the total time spent in the wood shavings area could be measured. Behaviours performed in the wood shavings area were analysed as a proportion of the total time spent there.

All birds were tested with all platform distances and water depths, even if they gave up crossing the water in earlier tests. This allowed statistical analyses of a full complement of longitudinal data which is likely to be more powerful than analyses of summary measures such as the maximum cost paid (distance/depth overcome) to get to the wood shavings area.

### Statistical Analysis

All measures were analysed by fitting Linear Mixed Models (LMMs) or Generalized Linear Mixed Models (GLMMs) using Residual Maximum Likelihood (REML) in Genstat (14^th^ edition 2011). Fixed effects included in all models were feed treatment (R, 2R, 3R), scheduling group (1,2) and apparatus (1,2), and for longitudinal data, test number (1,…,8) and 2 way interactions between test number and each of the other 3 factors. Random effects included in all models were pen (1,…,21) (i.e. bird triplet or pair), and bird (1,…,60) and, for longitudinal data, interactions of these with test number (1,…,8). There was little or no variation due to the 7 spatial blocks of 3 pens so this was not included in the random effects. All effects were fitted as factors (i.e. categorical classifications). As well as fitting models with feed treatment as a single factor with 3 levels, alternative models were investigated with a single contrast between each pair of feed treatments fitted before the feed treatment factor, in order to test specifically for differences between each pair of feed treatments.

The proportion of birds that reached the wood shavings area was investigated by fitting a GLMM with logit link function and binomially distributed errors [51], [52] to the binary outcome of whether the bird reached the wood shavings area or not. The highest cost each bird paid was analysed by fitting LMMs to the test number of the last test when a bird reached the wood shavings area. It was not possible to analyse the latency to reach the wood shavings area for all tests due to the large proportion (60%) of censored values. Latency to reach the wood shavings area for tests for which the bird did reach the wood shavings area only was analysed by fitting LMMs to natural log transformed data. The proportion of time spent in the wood shavings area (of 300 seconds opportunity to do so) for tests for which the bird did reach the wood shavings area only was analysed by fitting LMMs. The proportion of time spent foraging (scratching and pecking) while standing or sitting of the time spent in the wood shavings area was analysed by fitting LMMs to data transformed using the angular transformation [51]. Apart from the highest cost each bird paid, all of these analyses are of longitudinal data in which the effect of test number is included.

Statistical tests, with significance at the 5% level, were based on approximate *F* tests when these were available, referencing observed *F* statistics to the *F* distribution, but otherwise Wald tests were used in which the Wald statistic was compared to the *χ^2^*-distribution. In approximate *F* tests, denominator degrees of freedom are estimated and may not be whole numbers when fitted factors are not perfectly balanced/and or when effects are estimated from more than one level of the random hierarchy in the model. Test results given are based on sequential tests and factors are tested in different orders as appropriate to any imbalances between them in order to ensure that the results given are robust against test order, reporting results of each factor when tested last. For example, when analysing measurements from successful tests only, imbalances between feed treatment and test number occur due to loss of more data from feed treatment 3R and least from feed treatment R as test number increases. Therefore, tests for feed treatment are reported adjusting for test number, and for test number adjusting for feed treatment.

Estimated means and standard errors (SEs) from the LMMs and GLMMs are reported for the interaction between feed treatment and test number (averaged over the other fixed effects) and for main effects of feed treatment and test number when significant. In addition, in order to aid interpretation, back transformed means are reported for LMMs when applied to transformed data and for GLMMs. The dataset can be acquired from the PlosOne website as File S1.

## Results

### Reaching the wood shavings area

The highest cost each bird paid (‘Reservation price’ or ‘Maximum Price Paid’) was on average 2–3 times higher (*F*_2,17_ = 11.28, P<0.001) for R birds (mean ± SE test number = 6.30±0.64) than for 2R (mean ± SE test number = 2.28±0.65) and 3R (mean ± SE test number = 2.96±0.65) birds.

**Figure 3:**
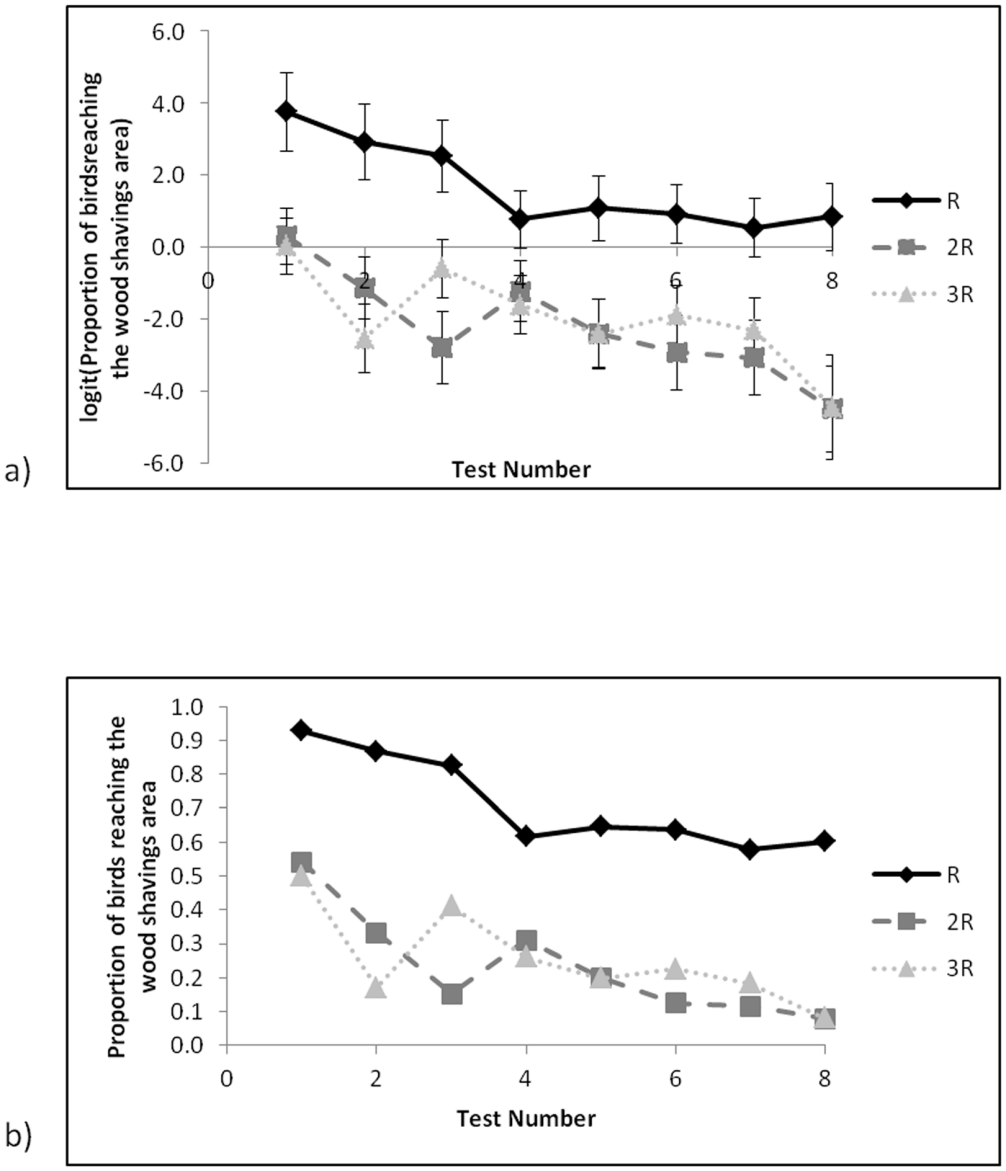
Proportion of birds reaching the wood shavings area across eight tests with increasing water runway length and depth for the three food treatments for a) means (±SE) estimated from GLMM on logit scale and b) back transformed to the proportion of birds reaching the wood shavings area.

There was an effect of feed treatment, with a larger proportion of birds fed R reaching the wood shavings area than birds fed 2R and 3R (χ^2^_2_ = 20.25, P<0.001, Fig 3) and there was a decrease in the proportion of birds from all feed treatments that reached the wood shavings area as test number increased (χ^2^_7_ = 25.45, P<0.001, Fig 3). There was no statistically significant interaction between test number and feed treatment.

**Figure 4:**
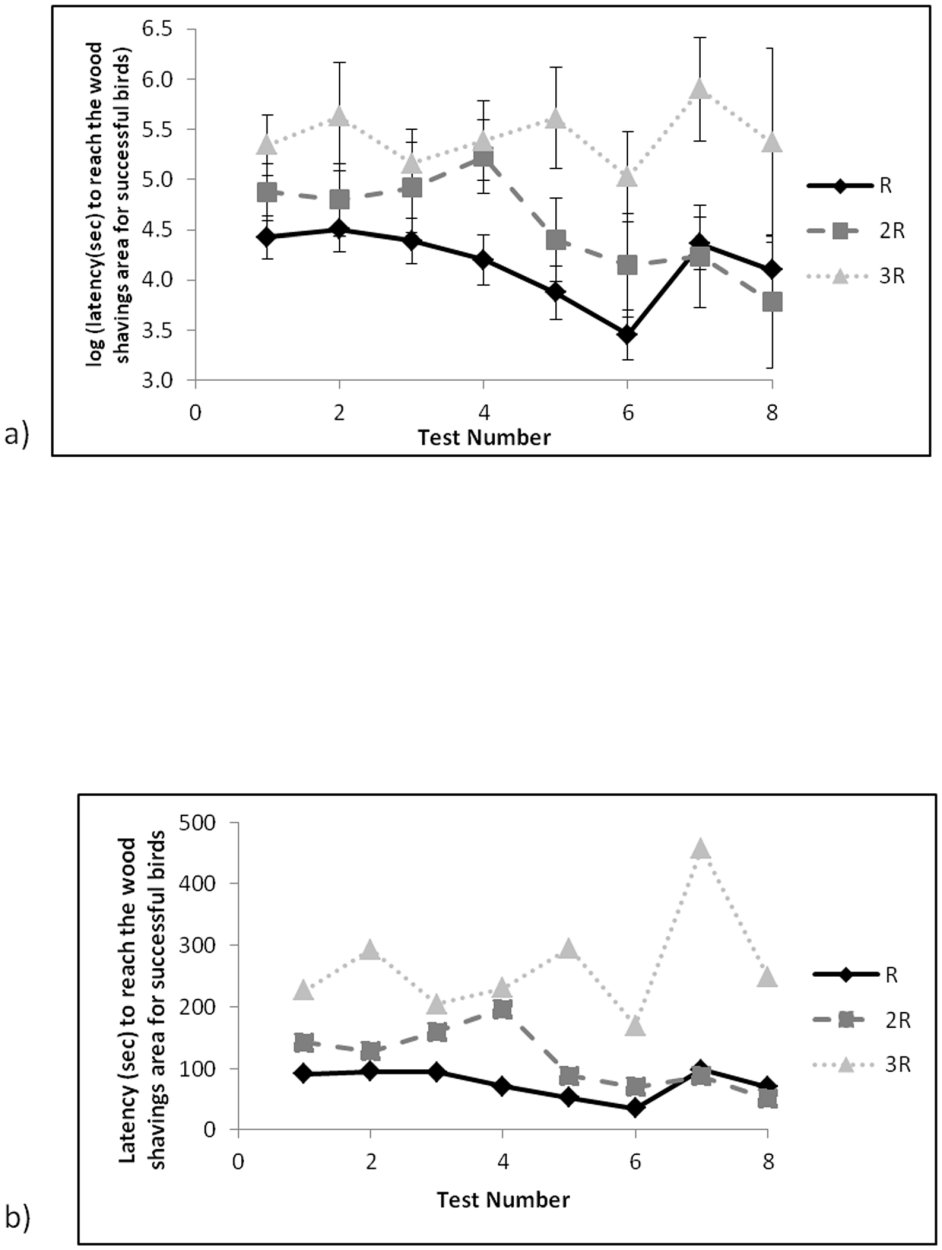
Latency to reach the wood shavings area for tests for which birds were successful across eight tests with increasing water runway length and depth for the three food treatments for a) means (±SE) estimated from LMM on log scale and b) back transformed to the latency to reach the wood shavings area.

For tests in which birds reached the wood shavings area only, on average, birds fed R were quickest to reach the wood shavings area, followed by birds fed 2R and then birds fed 3R (*F*_2,37_ = 11.55, P<0.001, Fig 4) and tests of specific contrasts suggested statistically significant differences in mean between all 3 feed treatments (R versus 2R *F*_1,33_ = 8.70, P = 0.006, R versus 3R *F*_1,37_ = 23.05, P<0.001, 2R vs 3R *F*_1,41_ = 5.02, P = 0.031). Test number was only marginally significant (*F*_7,49_ = 2.53, P = 0.027) and there was no statistically significant interaction between test number and feed treatment (Fig 4).

### Behaviour in the wood shavings area

**Figure 5:**
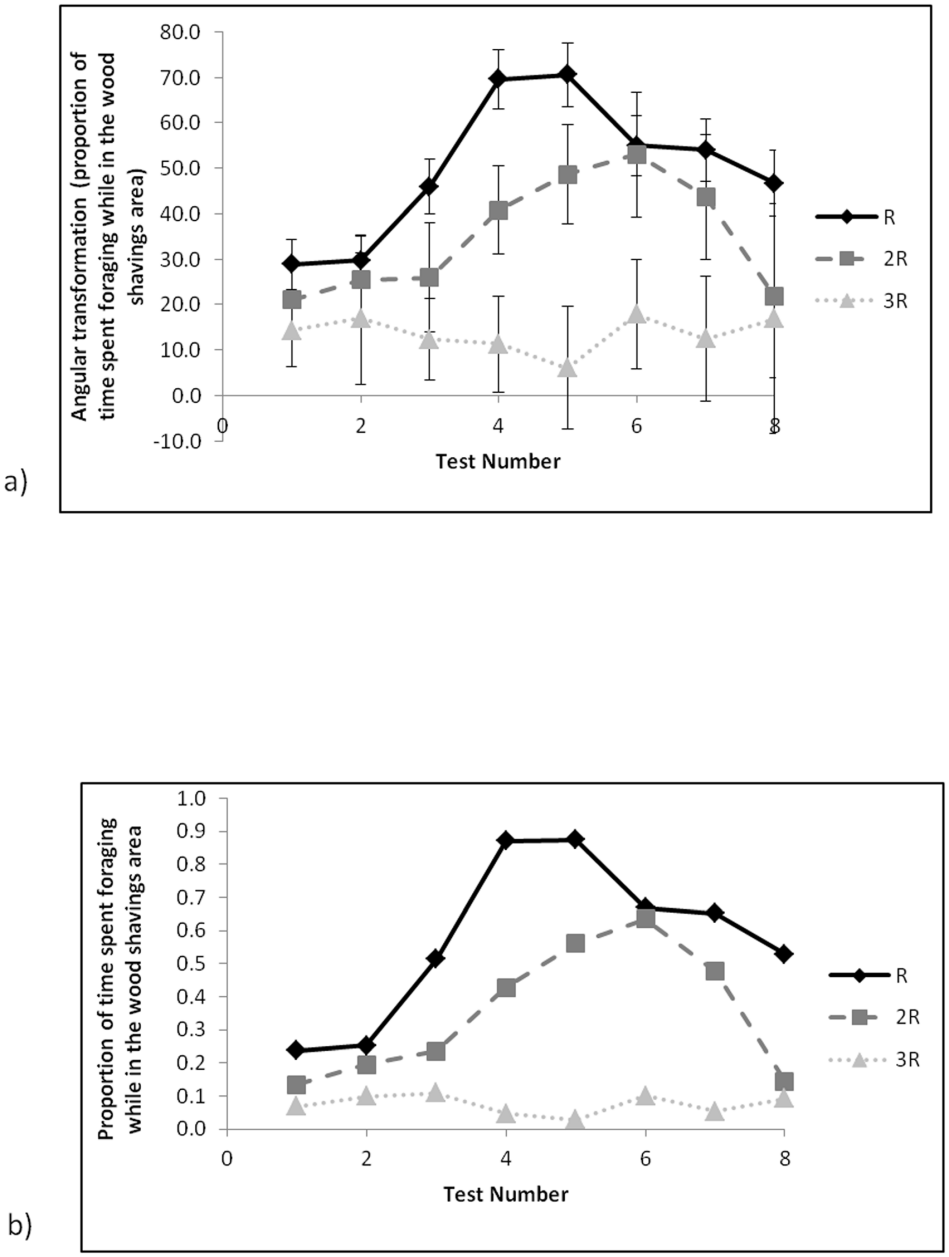
Proportion of time spent foraging while in the wood shavings area for tests for which birds were successful across eight tests with increasing water runway length and depth for the three food treatments for a) means (±SE) estimated from LMM on angular scale and b) back transformed to the proportion of time spent foraging while in the wood shavings area.

For tests in which birds reached the wood shavings area, there was no statistically significant effect of test number or feed treatment on the mean time spent in the wood shavings area; birds on average generally spent over 50% of the five minutes available there, ranging from 145±15 sec at the first test to 238±42 sec at the last test (mean ± SE estimated from LMM). However, when birds were in the wood shavings area, birds fed R spent the most time foraging whilst birds fed 3R spent the least time foraging (*F*_2,30_ = 18.54, P<0.001; Fig 5) and tests of specific contrasts suggested statistically significant differences in proportions between all 3 feed treatments (R versus 2R *F*_1,26_ = 14.95, P<0.001, R versus 3R *F*_1,30_ = 37.04, P<0.001, 2R vs 3R *F*_1,35_ = 7.12, P = 0.011). Overall, the proportion of time spent foraging (of the time spent in the wood shavings area) increased with test number and peaked at 0.49 at test number 5 and then decreased again (*F*_7,46_ = 7.56, P<0.001), with individual feed treatments peaking at 0.87 for R, 0.64 for 2R and 0.11 for 3R (back-transformed values; Fig 5). There was no statistically significant interaction between test number and feed treatment affecting the proportion of time spent foraging.

## Discussion

As expected, broiler breeders fed the commercially restricted food allowances (R) accessed the wood shavings area more at all levels of cost than the 2R and 3R birds, resulting in on average a higher Maximum Price Paid (Reservation Price) for access to the wood shavings area. Rate of decline in the proportion of birds reaching the wood shavings area appeared to be less steep for R birds but this effect was not statistically significant (no feed treatment by test interaction). The rate of change of consumption of a resource in response to increasing cost has been highlighted by some authors as a key measure of motivation [1], [53]. In this experiment, variation between birds, and the large treatment differences which already meant that a relatively low proportion of 2R and 3R birds visited the wood shavings area even at low cost made it difficult to detect this interaction. Unexpectedly, there was little difference between 2R and 3R birds in these measures, which might suggest that the welfare benefits in terms of reduced hunger of increasing rations from 2R to 3R are limited, in contrast to the welfare benefits of reducing hunger in R birds. Of birds that reached the wood shavings area, on average, restricted birds took less time to reach it and spent a greater proportion of their time there foraging; these behaviours were different between R and 2R, but also between 2R and 3R birds, so were more discriminatory of feeding level.

The low numbers of birds willing to cross the water in the 2R and 3R treatments is a potential weakness for using this technique in future studies when analysing measures that are only defined for birds that reach the wood shavings area. However, the range of treatments chosen here was large, and experimental diets designed to improve satiety which will be of relevance to the poultry industry are likely to be much closer to the R diet tested here. Comparison with this initial validation study will enable any improvements to be put into context.

All of these behavioural differences indicate that birds fed R were highly motivated to reach the wood shavings area in order to perform the food-searching (appetitive) component of foraging behaviour (pecking and scratching) even though they were never rewarded with food (allowed to progress to consummatory behaviour). Repeatedly failing to reward foraging behaviour in this location with a food reward might have been expected to result in extinction of the response [37]. In fact, R birds continued to access the wood shavings area, and on average the performance of foraging behaviour by R (and 2R) birds initially increased over successive tests, before eventually declining. In our study, cost increased over successive tests, so it is not possible to know whether this eventual decline was a result of extinction learning (i.e. that there was never any food), or because of the increasing cost of access. Interpretation of the time spent foraging is made more difficult by the fact that fewer birds reached the wood shavings area in later tests. However, for R birds, there were consistently ∼60–65% of them reaching the wood shavings area between tests 4 and 8, so the decline for these may indeed indicate extinction (although it was not statistically significant). In future studies, fewer tests could be used to reduce the possibility of extinction. There are different methods that can be used to present costs for access to a resource – we chose to progressively increase the cost over successive tests. However, costs can be presented, for example, in randomly determined orders or by progressively increasing then decreasing the cost of access. Different methods have their own advantages and disadvantages, for example, cost increases over time may become confounded with fatigue or boredom but random cost presentations may limit the Maximum Price that is tested, i.e. animals may be willing to work harder than the ‘highest cost’ given but additional ‘harder’ costs cannot be added to the end of the test [54]; how costs are presented to the animals should be carefully considered when designing a study to ensure questions of interest will be answered.

Our use of a non-food rewarded task adds to the evidence that commercial restriction levels result in chronic hunger in broiler breeders. Existing data that these birds have high feeding motivation (e.g. [41]) is open to the criticism that offering extra food changes the situation, and increases food motivation. High levels of foraging motivation, even in the absence of food, suggest that food is not ‘out of sight, out of mind’ for broiler breeders at commercial levels of food restriction [17].

Broilers (offspring of broiler breeders) which are fed *ad libitum* throughout their lives may have gait and movement problems due to their fast growth rates [55], [56]; however none of our broiler breeders exhibited any difficulties walking. This is most likely because all birds were reared on the recommended, restricted diets until 5 weeks of age, allowing for a slow, healthy growth until this point [21]. While birds fed 2R and 3R did grow faster than birds fed R, they were still more active than typical broilers in their home pens (L.M. Dixon, Pers. Obs.) throughout the trial which may also have helped prevent some fast growth related issues [55]. Therefore, the slower latencies for 2R and 3R birds to reach the wood shavings area cannot simply be due to difficulties walking in heavier birds. It is well established that broiler breeders which are restricted (R) are more active than those which are more generously fed [25]–[29]. Anecdotally, R birds in our study appeared more active in the apparatus, and it is possible that this explains some aspects of the behavioural differences between the treatments. However, the increased foraging behaviour, and tolerance of deeper water in R birds suggests that as well as being more active, they were also highly motivated to forage.

In future studies, we hope to apply the water runway method to novel diets designed to improve satiety. It will be interesting to compare this method to more conventional home pen behavioural measures, such as time spent resting, foraging and performing abnormal or stereotypic behaviour patterns [27], [29]. Additionally comparing these results to tests in which food rewards are offered [29], [30], [31], [32] may help to quantify the changes in motivation that occur due to feedback from the food and may help present a complete picture of hunger in broiler breeders. Various physiological measures of the welfare consequences of hunger have been proposed (e.g. [33]), although the question of validation and comparison between food types remain problematic (see discussion in [35]). Recent work to develop measures that relate directly to the neural circuits in the basal hypothalamus that control food intake [57] shows promise.

In the present study, we validated a test using a natural, variable cost to measure the motivation to perform appetitive behaviour by comparing different feed treatments. There are a number of practices in the broiler breeder industry that have been proposed as ways of reducing the hunger experienced due to restricted feeding. These include modifications of feeding methods; such as adding fibre to the diet [29], [32], scatter feeding to increase feeding time and encourage foraging [58], feeding multiple small meals [58] and skip-a-day feeding [59]; and genetic changes, for example by using slow growing or dwarf strains [60], [61], or by genetic selection to change the shape of the growth curve [62]. The foraging motivation test used here has potential to be applied alongside other behavioural and physiological measures to determine whether any of these practices result in reduced foraging motivation and thus in reduced hunger and improved welfare.

## Conclusions

In conclusion, broiler breeders fed commercially restricted food allowances are more motivated to access an area of wood shavings where they can forage than birds fed twice or three times that amount. This indicates that these birds are hungry and as a result have decreased welfare. In future, the methods described here can be used to assess different broiler breeder practices and strains to attempt to decrease hunger and improve welfare in the broiler breeder industry.

## Supporting Information

File S1 **Raw data.**
(XLSX)

## References

[1] Dawkins MS (1990) From an animal's point of view: motivation, fitness and animal welfare. Behaviour and Brain Science 13: 1–61.

[2] Duncan IJH (1996) Animal welfare defined in terms of feelings. Acta Agriculturae Scandinavica Section A-Animal Science S27: 29–35.

[3] Duncan IJH (2005) Science-based assessment of animal welfare: farm animals. Revue Scientifique et Technique-Office International des Epizooties 24: 483–492. 16358502

[4] Dawkins MS (2006) Through animal eyes: What behaviour tells us. Applied Animal Behaviour Science 100: 4–10 10.1016/j.applanim.2006.04.010

[5] Dawkins MS (2008) The science of animal suffering. Ethology 114: 937–945.

[6] Mendl M, Burman OHP, Paul ES (2010) An integrative and functional framework for the study of animal emotion and mood. Proceedings of the Royal Society of London B: Biological Sciences 277: 2895–2904. 10.1098/rspb.2010.0303 PMC2982018 20685706

[7] Duncan IJH (1992) Measuring preferences and the strength of preference. Poultry Science 71: 658–663. 10.3382/ps.0710658 1594518

[8] Dawkins MS (1983) Battery hens name their price: Consumer demand theory and the measurement of ethological ‘needs’. Animal Behaviour 31: 1195–1205 10.1016/S0003-3472(83)80026-8

[9] Mason GJ, Cooper JJ, Clarebourgh C (2001) Frustrations of fur-farmed mink. Nature 410: 35–36. 11242031 10.1038/35065157

[10] Seaman S, Waran NK, Mason GJ, D'Eath RB (2008) Animal economics: assessing the motivation of female laboratory rabbits to reach a platform, social contact or food. Animal Behaviour 75: 31–42.

[11] Kirkden RD, Pajor EA (2006) Using preference, motivation and aversion tests to ask scientific questions about animals' feelings. Applied Animal Behaviour Science 100: 29–47 10.1016/j.applanim.2006.04.009

[12] Jensen MB, Pedersen LJ (2008) Using motivation tests to assess ethological needs and preferences. Applied Animal Behaviour Science 113: 340–356 10.1016/j.applanim.2008.02.001

[13] Fraser D, Matthews LR (1997) Preference and motivational testing. In: Appleby MC, Hughes BO, editors. Animal Welfare. Wallingford: CAB International. pp. 159–173.

[14] Dixon LM, Sandilands V, Bateson M, Brocklehurst S, Tolkamp BJ, et al (2013) Conditioned place preference or aversion as animal welfare assessment tools: Limitations in their application. Applied Animal Behaviour Science 148: 164–176.

[15] Faure JM, Lagadic H (1994) Elasticity of demand for food and sand in laying hens subjected to variable wind speed. Applied Animal Behaviour Science 42: 49–59 10.1016/0168-1591(94)90006-X

[16] Olsson IAS, Keeling LJ (2002) The push-door for measuring motivation in hens: laying hens are motivated to perch at night. Animal Welfare 11: 11–19.

[17] Warburton H, Mason G (2003) Is out of sight out of mind? The effects of resource cues on motivation in mink, *Mustela vison* . Animal Behaviour 65: 755–762.

[18] Renema RA, Robinson FE (2004) Defining normal: comparison of feed restriction and full feeding of female broiler breeders. World's Poultry Science Journal 60: 508–522.

[19] Savory CJ, Hocking PM, Mann JS, Maxwell MH (1996) Is broiler breeder welfare improved by using qualitative rather than quantitative food restriction ti limit growth rate? Animal Behaviour 5: 105–127.

[20] de Jong IC, van Voorst S, Ehlhardt DA, Blokhuis HJ (2002) Effects of restricted feeding on physiological stress parameters in growing broiler breeders. British Poultry Science 43: 157–168. 10.1080/00071660120121355 12047078

[21] Mench JA (2002) Broiler breeders: feed restriction and welfare. World's Poultry Science Association 58: 23–29.

[22] D'Eath RB, Tolkamp BJ, Kyriazakis I, Lawrence AB (2009) “Freedom from hunger” and preventing obesity: the animal welfare implications of reducing food quantity or quality. Animal Behaviour 77: 275–288 10.1016/j.anbehav.2008.10.028

[23] Decuypere E, Bruggeman V, Everaert N, Li Y, Boonen R, et al (2010) The Broiler Breeder Paradox: ethical, genetic and physiological perspectives, and suggestions for solutions. British Poultry Science 51: 569–579. 10.1080/00071668.2010.519121 21058058

[24] Dawkins MS (2004) Using behaviour to assess animal welfare. Animal Welfare 13: S3–7.

[25] Hocking PM, Maxwell MH, Robertson GW, Mitchell MA (2001) Welfare assessment of modified rearing programmes for broiler breeders. British Poultry Science 42: 424–432. 10.1080/00071660120070677 11572616

[26] Savory CJ, Seawright E, Watson A (1992) Stereotyped behavior in broiler breeders in relation to husbandry and opioid receptor blockade. Applied Animal Behaviour Science 32: 349–360.

[27] Savory CJ, Maros K (1993) Influence of degree of food restriction, age and time of day on behavior of broiler breeder chickens. Behavioural Processes 29: 179–190. 24895933 10.1016/0376-6357(93)90122-8

[28] Hocking PM, Maxwell MH, Mitchell MA (1996) Relationships between the degree of food restriction and welfare indices in broiler breeder females. British Poultry Science 37: 263–278. 10.1080/00071669608417858 8773836

[29] Sandilands V, Tolkamp BJ, Kyriazakis I (2005) Behaviour of food restricted broilers during rearing and lay: effects of an alternative feeding method. Physiology and Behaviour 85: 115–123. 10.1016/j.physbeh.2005.03.001 15878603

[30] Savory CJ, Maros K, Rutter SM (1993) Assessment of hunger in growing broiler breeders in relation to a commercial restricted feeding programme. Animal Welfare 2: 131–152.

[31] Savory CJ, Lariviere JM (2000) Effects of qualitative and quantitative food restriction treatments on feeding motivational state and general activity level of growing broiler breeders. Applied Animal Behaviour Science 69: 135–147. 10906398 10.1016/s0168-1591(00)00123-4

[32] Sandilands V, Tolkamp BJ, Savory CJ, Kyriazakis I (2006) Behaviour and welfare of broiler breeders fed qualitatively restricted diets during rearing: are there viable alternatives to quantitative restriction? Applied Animal Behaviour Science 96: 53–67.

[33] de Jong IC, van Voorst S, Blokhuis HJ (2003) Parameters for quantification of hunger in broiler breeders. Physiology and Behaviour 78: 773–783. 10.1016/s0031-9384(03)00058-1 12782235

[34] de Jong IC, Enting H, van Voorst S, Ruesink EW, Blokhuis HJ (2005) Do low density diets improve broiler breeder welfare during rearing and laying? Poultry Science 84: 194–203. 10.1093/ps/84.2.194 15742954

[35] D'Eath RB, Tolkamp BJ, Kyriazakis I, Lawrence AB (2009) Freedom from hunger and preventing obesity: the animal welfare implications of reducing food quantity or quality. Animal Behaviour 77: 275–288 10.1016/j.anbehav.2008.10.028

[36] Day JEL, Kyriazakis I, Lawrence AB (1996) The use of a second-order schedule to measure feeding motivation in the pig. Applied Animal Behaviour Science 50: 15–31.

[37] Mills DS, Marchant-Forde JN, McGreevy PD, Morton DB, Nicol CJ, et al. (2010) Encyclopedia of Applied Animal Behaviour. Wallingford: CAB International.

[38] Berridge KC (2004) Motivation concepts in behavioral neuroscience. Physiology and Behaviour 81: 179–209. 10.1016/j.physbeh.2004.02.004 15159167

[39] Toates FM (1998) Motivation. In: Toates FM, editor. Control of Behaviour. Singapore: Springer-Verlag. pp. 15–45.

[40] McFarland DJ (1974) Time sharing as a behavioural phenomenon. Advances in the Study of Behaviour 5: 201–225.

[41] Savory CJ, Maros K, Rutter SM (1993) Assessment of hunger in growing broiler breeders in relation to a commercial restricted feeding programme. Animal Welfare 2: 131–152.

[42] Savory CJ, Lariviere JM (2000) Effects of qualitative and quantitative food restriction treatments on feeding motivational state and general activity level of growing broiler breeders. Applied Animal Behaviour Science 69: 135–147. 10906398 10.1016/s0168-1591(00)00123-4

[43] Cooper JJ, Appleby MC (1995) Nesting behaviour of hens: Effects of experience on motivation. Applied Animal Behaviour Science 42: 283–295 10.1016/0168-1591(94)00543-N

[44] Verbeek E, Waas JR, McLeay L, Matthews LR (2011) Measurement of feeding motivation in sheep and the effects of food restriction. Applied Animal Behaviour Science 132: 121–130 10.1016/j.applanim.2011.03.014

[45] Cooper JJ, Appleby MC (1994) The use of aversive barriers to quantify nesting motivation in domestic hens. Proc Symp Modified Cages 11–24.

[46] Weeks CA (2001) Development of a newmethod for quantitatively assessing lameness in broilers. British Poultry Science 42: S79–S80.

[47] Weeks CA, Knowles TG, Gordon RG, Kerr AE, Peyton ST, et al (2002) New method for objectively assessing lameness in broiler chickens. Veterinary Record 151: 762–764. 12521248

[48] Aviagen (2013) Ross 308 Parentstock Management Handbook 2013. Available: http://en.aviagen.com/assets/Tech_Center/Ross_PS/Ross_PS_Handbook_2013_i-r1.pdf. Accessed 2014 Jul 8.

[49] DEFRA (2006) The structure of the United Kingdom poultry industry: Commercial Poultry Sector. Available: http://labourproviders.org.uk/wp-content/uploads/2012/11/Poultry_sector.pdf. Accessed 2014 Jul 8.

[50] Buckley LA, Sandilands V, Tolkamp BJ, D'Eath RB (2011) Quantifying hungry broiler breeder dietary preferences using a closed economy T-maze task. Applied Animal Behaviour Science 133: 216–227 10.1016/j.applanim.2011.06.003

[51] Collett D (1999) Modelling Binary Data. London: Chapman & Hall.

[52] Brown H, Prescott R (1999) Applied Mixed Models in Medicine. New York: J. Wiley & Sons.

[53] Matthews LR, Ladewig J (1994) Environmental requirements of pigs measured by behavioural demand functions. Animal Behaviour 47: 713–719.

[54] Asher L, Kirkden RD, Bateson M (2009) An empirical investigation of two assumptions of motivation testing in captive starlings (Sturnus vulgaris): Do animals have an energy budget to ‘spend’? and does cost reduce demand? Applied Animal Behaviour Science 118: 152–160 10.1016/j.applanim.2009.02.029

[55] Bessei W (2006) Welfare of broilers: a review. World's Poultry Science Journal 62: 455–466.

[56] Kestin SC, Knowles TG, Tinch AE, Gregory NG (1992) Prevalence of leg weakness in broiler chickens and its relationship with genotype. Veterinary Record 131: 190–194. 1441174 10.1136/vr.131.9.190

[57] Dunn IC, Wilson PW, Smulders TV, Sandilands V, D'Eath RB, et al (2013) Hypothalamic agouti-related protein expression is affected by both acute and chronic experience of food restriction and re-feeding in chickens. Journal of Neuroendocrinology 25: 920–928. 23957836 10.1111/jne.12088

[58] de Jong IC, Fillerup M, Blokhuis HJ (2005) Effect of scattered feeding and feeding twice a day during rearing on indicators of hunger and frustration in broiler breeders. Applied Animal Behaviour Science 92: 61–76.

[59] Mench JA (1991) Research note: Feed restriction in broiler breeders causes a persistent elevation in corticosterone secretion that is modulated by dietary tryptophan. Poultry Science 70: 2547–2550. 10.3382/ps.0702547 1784577

[60] Proudfoot FG, Hulan HA, McRae KB (1984) Effects of photoperiod, light intensity and feed restriction on the performance of dwarf and normal poultry meat genotypes. Canadian Journal of Animal Science 64: 759–768.

[61] Jones EKM, Zaczek V, MacLeod M, Hocking PM (2004) Genotype, dietary manipulation and food allocation affect indices of welfare in broiler breeders. British Poultry Science 45: 725–737. 10.1080/00071660400014226 15697011

[62] Dawkins MS, Layton R (2012) Breeding for better welfare: genetic goals for broiler chickens and their parents. Animal Welfare 21: 147–155.

